# A review of *Central Nervous System Metastases: Diagnosis and Treatment*, First Edition, 2020

**DOI:** 10.1093/noajnl/vdaa102

**Published:** 2020-08-26

**Authors:** Jeffrey A Zuccato, Philip J O’Halloran, Gelareh Zadeh

**Affiliations:** 1 Division of Neurosurgery, University Health Network, University of Toronto, Toronto, Ontario, Canada; 2 MacFeeters-Hamilton Center for Neuro-Oncology, Princess Margaret Cancer Center, Toronto, Ontario, Canada; 3 Royal College of Surgeons in Ireland, Dublin, Ireland

**Keywords:** brain metastases, cancer, central nervous system, textbook review, spinal metastases

Metastases from primary solid organ cancers are the most common central nervous system (CNS) tumor type and their incidence is increasing with improved control of systemic disease. Multidisciplinary treatment with personalized therapeutic strategies is required for these patients in order to optimally balance oncological and quality of life outcomes. This comprehensive textbook brings together experts across many fields to produce a concise resource on all important and current topics regarding the care of CNS metastasis patients.

As metastases comprise the majority of central nervous system (CNS) tumors and are increasing in incidence due to enhanced treatment strategies that improve systemic cancer control, management is becoming increasingly complex and personalized.^1–3^ Therefore, a multidisciplinary resource summarizing the contemporary approaches to diagnosis and management of these lesions is very timely. Accordingly, in May of this year, Springer published a textbook entitled *Central Nervous System Metastases: Diagnosis and Treatment* that is a comprehensive multi-disciplinary resource on the management of CNS metastases.^4^

The editors Rohan Ramakrishna (Director of Brain Metastases Program, Department of Neurological Surgery), Rajiv S. Magge (Department of Neurology), Ali A. Baaj (Department of Neurological Surgery), and Jonathan P.S. Knisely (Department of Radiation Oncology) from the New York-Presbyterian Hospital/Weill Cornell Medicine in New York, New York, United States worked with over 130 contributors to create this concise 718-page, 7-part textbook. Contributors included leaders with clinical and research expertise in neurosurgery, radiation oncology, medical oncology, medicine, neurology, neuroradiology, plastic surgery, pain medicine, palliative care, neuropsychology, psychiatry, physical medicine & rehabilitation, and neuropathology.

## Discussion of Content

### Part I: Fundamentals

The section orients the reader to the epidemiology of CNS metastases and summarizes the mechanistic biology underlying the process of cancer metastasizing to the CNS. Implementation of clinically relevant translational models is highlighted as a significant component in the development of novel targeted therapies. Important principles of neuropathological diagnosis are described along with molecular alterations and corresponding targeted treatments. Finally, the utilization of clinical decision-making tools and advanced imaging techniques in the care of these patients is described. Overall, the initial section of the textbook succinctly introduces the reader to the field and provides context for the following sections on the evaluation and management of CNS metastatic disease.

### Part II: Evaluation of CNS Metastasis

Here, the reader encounters a summary of the clinical manifestations of CNS metastases and a concise outline of the systemic therapies available for CNS metastases by cancer type. Dedicated chapters describe additional considerations for the evaluation and management of leptomeningeal disease, mood disorders, and pediatric patients. This section also includes chapters focusing on paraneoplastic neurological disorders (PNDs) and cerebrovascular lesions that can occur in cancer as additional CNS manifestations. Not only does this section provide great detail regarding the evaluation of CNS metastases, the all-encompassing nature of the textbook is highlighted here by the inclusion of content regarding other CNS complications of cancer like PNDs that may develop in cancer patients without CNS metastases.

### Part III: Radiation Therapy of CNS Metastases

The radiotherapy section of this book nicely summarizes the physics and biology foundations for radiation treatments that provide context for the understanding of the various options used to manage CNS metastases. In particular, the roles for whole-brain therapy (WBRT) and stereotactic radiosurgery (SRS) along with approaches to reduce the side effects and complications of radiotherapy are discussed. Finally, the dilemma of diagnosing radiation necrosis or pseudoprogression from tumor recurrence as well as the use of reirradiation after recurrence are outlined. Within this section, the authors very effectively distill down the evidence for each therapeutic option with clear and comprehensive tables containing relevant studies and trials. Clinical case reports were nicely used throughout to highlight the role for various radiotherapy approaches in both important and challenging scenarios.

### Part IV: Surgical Treatment of Brain Metastasis

The content of the textbook then nicely flows into the role for surgical resection of brain metastases and approaches to deciding between surgery and definitive radiotherapy in various clinical settings. Strategies to improve surgical outcomes are discussed including the utilization of advanced imaging techniques along with intraoperative mapping. New techniques are outlined as well ranging from intraoperative brachytherapy to laser interstitial thermal therapy (LITT) as a minimally invasive technique to ablate tumors. Finally, important considerations in managing cranial wound complications are summarized. Altogether, this section outlines how surgical treatment fits within the framework of multi-disciplinary care for brain metastasis patients and describes exciting new minimally invasive strategies with the evidence for them.

### Part V: Spinal Metastases: Foundations

This section expands on the fundamental CNS metastasis content in Part I with specific foundational principles related to spinal lesions from clinical factors to imaging and treatment considerations. Additionally, readers will encounter detail regarding bone metabolism and antiresorptive treatments for bony metastases, principles for managing spinal cord compression, and the application of advanced imaging techniques to spinal metastases. This section ends with a discussion of algorithms that guide decision-making regarding the treatment of spinal metastasis patients which effectively consolidates the content here for the reader and highlights important concepts.

### Part VI: Surgical Treatment of Spinal Metastases

Part VI nicely complements Part IV that focuses on surgical management of brain metastases. After an introduction to spinal biomechanics, spinal stabilization techniques are summarized. Indications for surgery versus radiotherapy are discussed with specific considerations for the roles of vertebrectomy and intraoperative radiotherapy. Minimally invasive approaches are outlined including separation surgery with concomitant stereotactic body radiotherapy (SBRT), minimally invasive decompression and fixation, vertebroplasty/kyphoplasty, and LITT. Additional content concisely summarizes post-surgical complications with focus on the management of wound healing issues. Overall, this section provides a detailed description of the role for surgery with effective use of diagrams depicting biomechanical properties and radiographical images showing important fixation principles.

### Part VII: Pain

Finally, the authors discuss approaches to managing CNS metastasis pain including a well-organized outline of all the available pharmacological agents along with resective and ablative spinal procedures, intrathecal agents, and spinal cord stimulation as approaches to manage refractory pain. Finally, palliative and supportive care strategies along with available alternative complementary and integrative therapies are discussed. As with other sections, this section continues to be a comprehensive guide and the inclusion of alternatives treatments that are being increasingly considering by patients is notable.

## Conclusion

This textbook is a comprehensive and well laid out resource that provides succinct descriptions of all topics relevant to the management of CNS metastasis patients from classical approaches to novel treatment strategies. Leaders in the field from a wide range of disciplines have come together to create an invaluable resource for multidisciplinary providers managing CNS metastasis patients as well as for researchers aiming to improve their care.

## Notes

**Authorship statement.** Manuscript preparation and revision (J.A.Z., P.J.O., G.Z.).
